# The early bird gets the return: The benefits of publishing your data sooner

**DOI:** 10.1002/ece3.7853

**Published:** 2021-07-06

**Authors:** Christopher J. Lortie

**Affiliations:** ^1^ Department of Biology York University Toronto ON Canada

## Abstract

The benefits of publishing your data sooner versus later in Ecology and Evolution.

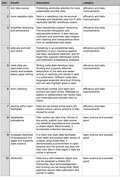

## 1 INTRODUCTION

Data are a fundamental component of the scientific process. Data can inform ideas, are one of many critical outputs of experimentation, and are the substrate of most traditional scientific reporting formats in journals in disciplines such as Ecology and Evolution. It has also been proposed that papers presenting more data and relatively more diverse data are more cited in Ecology and Evolution (Fox et al., 2016). Data and theory need not, however, compete for relevance in any scientific disciplines including ecology (Betts et al., 2021; Fagerström, 1987; Shrader‐Frechette & McCoy, 1990; Spaargaren et al., 2009). In many respects, computation and work with data can iteratively feedback to theory development and novel ideas including new experiments (Markowetz, 2017). Data, when aggregated or well articulated, can also directly and collectively inform best practices in fields such as restoration ecology in addition to the peer‐reviewed publications and engender transdisciplinary solutions that involve connecting the dots through data between disciplines (Ladouceur & Shackelford, 2021). Data are thus a form of currency in many disciplines (Fan et al., 2012) but can also become a bottleneck if sharing is delayed (Westoby et al., 2021), have poorly described metadata (Madin et al., 2007), or if there are limited incentives within the scientific culture (Michener, 2015a). Consequently, it is reasonable to propose that one should consider sharing data sooner versus later to remove or reduce the impact of these bottlenecks, particularly temporal ones, on the modern scientific process that now includes scientific communication, synthesis, and open publication as powerful final steps to connect with societal needs (Cooke et al., 2017). Full open‐access journals such as Ecology and Evolution are an ideal partner that embody these open science principles including transparency and effective, timely sharing.

Hence, the following strategy is reasonable in many contexts. Publish your data when the phase of scientific collection/compilation is complete and before you begin writing the associated preprint, report, or paper. Share the data and metadata openly in a data repository with a DOI (digital online identifier to enable citation and permanence) and versioning (i.e., updated versions are tracked and listed) (Kenall et al., 2014; Michener, 2015b). This is not an entirely novel proposal, but it is also not the norm in the historical or even contemporary culture of many scientific disciplines including Ecology and Evolution (Allen & Mehler, 2019). These two subdisciplines of science are leaders in the advancement of many open science endeavors (Hampton et al., 2015), and big data in ecology in terms of scope and diversity of evidence thresholds have been crossed (Hampton et al., 2013). In Ecology and Evolution, the relative importance of this transparency was proposed a number of years ago to better enable fair and critical assessment of the credibility of work and to provide the means to moderate interpretations of the relative importance of a study and its data (Shaw et al., 2016). The legacy and value inherent in data for reuse including synthesis and new ways were also pioneered in these fields (Whitlock et al., 2010). Nonetheless, sharing sooner enables more rapid and effective discovery, and dramatic knowledge and process changes can happen. The COVID‐19 pandemic has highlighted the incredible power of sharing data, simple summary statistics, and all findings extremely rapidly as collected or even in real time (George et al., 2020; Saitz & Schwitzer, 2020). The public and scientific community at large used these resources to engage in open, novel data‐driven science and evidence‐informed decision making (Agley, 2020; Devine et al., 2020), and these changes also innovated global learning (Lashley et al., 2020). In many other disciplines, we can adopt these lessons of being open and more rapid in sharing for both societal good and better science. Ecology and Evolution are perfectly poised to embrace these changes in sharing not only publications that describe “bright spots” in the Anthropocene (Bennett et al., 2016) but data rapidly and openly archived for consumption, review, and reuse. This will enable new syntheses and opportunities to synthesize data associated with global change for many issues. Direct and proximate benefits to the primary researcher are proposed here, and this simple change in timing (when reasonable and viable based on your research, laboratory, and career needs) will shift the culture of practice.

## 2 BENEFITS

Global and societal benefits aside (and these are not trivial as described above), publishing data when the experiment, synthesis, or primary scientific inquiry process is complete benefits the individual researcher and collaborators in at least ten critical and distinct ways (Table 1). There are two direct classes of benefits including (a) efficiency and data improvements (quality, access, integrity, provenance, etc.) and (b) team science with societal good implications (Gabrys et al., 2016). The concept of efficiency assumes analyses, visualization, writing, and peer‐review need seamless and likely repeat access to the data and metadata as we work. Workflows can be simple or complex, but it is reasonable to propose that we must access and read (reread) our data reproducibly to explore ideas—even as an individual researcher or team with our own datasets (Wilson et al., 2017). Data improvements are a more general concept predicated upon the assumption that data should be preserved in their most truthful form and then cleaned, tidied, and explored through computational tools such as R programming language (Grolemund & Wickham, 2016; Wickham, 2014). Ecoinformatic thinking is a component of most Ecology and Evolution, and it is not coupled to the size or complexity of a dataset or team (Michener & Jones, 2012). Team science and societal benefits are the positive outcomes associated with supporting transparency in all the steps of our work including “works‐in‐progress” to highlight the relative importance of process versus singular product thinking for many disciplines including Ecology and Evolution. Sharing work sooner supports a new more inclusive culture. Full stop. Sooner enables deeper opportunities for connection with others at multiple steps in addition to review of papers (Glynn et al., 2017). This bigger picture thinking is similar to the concept of registered reports (Nosek & Lakens, 2014) and other tools that we can use to signal intent and share ideas even if we are not at the final stage of writing (if that is one of the goals of a project but there can be others). Both categories of benefits thus directly improve the research process for individuals, but the scale of benefits and implications varies by the specific benefit.

**TABLE 1:** A list of proposed benefits to publishing data when the data collection process is complete. The benefits of publishing data prior to report or paper writing are developed, and two categories of benefit are proposed

Item	Benefit	Description	Category
1	Tidy data sooner	Publishing promotes attention to more presentable and tidy data	Efficiency and data improvements
2	More readable data	Data in a repository can be sourced remotely and iteratively read into R with replicable QA/WC workflows coded	Efficiency and data improvements
3	Simplifies versioning and provenance	Data repositories support versioning. This ensures transparent and reproducible science. It also reduces confusion and promotes data integrity with cleaning and manipulations done from an established data asset	Efficiency and data improvements
4	Data are archived and online	Publishing in an established data repository is your insurance against lost data, accidental deletions, and for larger files supports distributed online and potentially collaborative analyses	Efficiency and data improvements
5	Metadata are described more clearly and enable easier paper writing	Writing metadata develops clear thinking and supports effective description of the data and easier writing of methods and results if used in a publication. Different metadata languages engender structural thinking and can also highlight gaps in data.	Efficiency and data improvements
6	Error checking	Individuals outside your team and authors can catch errors. Referees on papers or stakeholders can review data and metadata and provide input on clarity	Efficiency and data improvements
7	Sharing within team facilitated	Data can be stored online and a link shared versus various versions of files shared by email	Efficiency and data improvements
8	Establishes precedence	Peer review can take time. Similar to preprints, publish your data sooner can establish precedence and provide a citable object. More broadly, it accelerates collective discovery	Team science and societal good
9	Increases likelihood of acceptance of a paper and supports open science	It is likely that open data facilitate more rapid and positive peer review of a paper using those data. It demonstrates a commitment to open science, and the authors can also cite their own data in their paper if data are online in advance	Team science and societal good
10	Attribution	Data are a valid research object and can be assigned a citable DOI. Authorship, land acknowledgement, and funding can be recognized. Most agencies require data publication, and sooner is better	Team science and societal good

The first seven proposed benefits all stem from the anchor that data archived online, accessed remotely, and reasonably well formatted with metadata catalyze positive opportunities for individuals and teams to both avoid errors and adopt better data management and open science practices (Table 1). Publishing sooner increases the likelihood that data will more presentable, more readable, and better tracked. This is a corollary of the Hawthorne effect from the social sciences that proposes positive outcomes emerge from interventions including observation or participation effects (Mayo, 1949; McCambridge et al., 2014). Furthermore, code can be used to read data remotely, check for errors, do manipulations, and link data to other resources while keeping the original data intact (Kumuthini et al., 2020). This also enables reproducible team science and replication (Fanelli, 2018; Reed, 2018). Data repositories support versioning so tracked updates are also possible, and this promotes more transparent and accountable science (including an opportunity to revisit previous instances). Data in a repository online are also your insurance policy against lost data, accidental deletions, and propagation of these errors (Buneman et al., 2002), and for larger files, online can resolve storage challenges locally. Publishing data in most data repositories in Ecology and Evolution also ensures that metadata are described appropriately and adhere to metadata standards for a specific metadata language required by many member nodes within the DataOne network for instance (a federation of data repositories) (Michener et al., 2012). Writing metadata (i.e., documenting key/all aspects of data) can also become the stepping stone for the methods section in a paper. Error checking is dramatically improved through relatively longer review and access periods, additional checks by yourself and the team, and finally by publishing the data prior to peer review of the paper, you provide an opportunity for independent checks of the data by referees if they so choose (it happens). Stakeholders can also engage with the evidence, that is, data, if openly archived, and this can precipitate novel insights. The final item directly associated with efficiency is that by sharing data online, you enable the capacity for collaboration via a shared linked versus interactions and changes to a file communicated via email. Less email and tracking of files sent back and forth are a massive gain in time and integrity in your work. A brief caveat is worth mentioning. Many of these benefits of efficiency and data integrity (but not necessarily preservation) can be accrued through remote storage, privately until the team is prepared for full and open global access. Many repositories and other tools support private sharing that still provide the capacity for remote storage, easy access, and versioning. This is a viable consideration depending on the circumstances, and with some forward thinking, this approach can still set one up for much easier and more rapid final data publication.

The final three proposed benefits link more directly to positive outcomes for team science and societal good. Peer review of papers can take time (Csiszar, 2016). Similar to preprints, publishing your data sooner can establish precedence and provide a citable object. There is also the capacity for establishing ideas sooner through not just preprints but also via data or code to generate additional interest and use (estimated through citations or altmetrics) in the final paper (from the project if that is the goal) (Eysenbach, 2006; Fu & Hughey, 2019; Serghiou & Ioannidis, 2018; Shuai et al., 2012). More broadly, it accelerates collective discovery. More specifically, open data are more cited (Piwowar & Vision, 2013) and sooner extends the citation window. Sharing an idea in different forms such as a paper (written word), data, code, or visually through slide decks also promotes a more accessible and diverse set of opportunities for people to engage with concepts. Next, this is more speculative and cultural, and sharing your data sooner sends a signal that you are committed to open science right the start. That is not to say that one should be disadvantaged if they cannot publish data before a paper, but that when possible, sharing and at least getting the data accessibility statement sorted for the submission of papers signal goodwill to editors and referees that the community can or will be able access the supporting evidence directly. Researchers can also cite their own data in the paper if available in advance (Zhao et al., 2018). Finally, data are a valid research object and an opportunity to acknowledge and provide attribution. These can be contributions and recognition associated with the data collection that may not necessarily be reflected in the authorship of the publication or report (i.e., paid technicians or others contributed to this component). Unfortunately, it is also not a given that data or evidence collected will always become a preprint, paper, or written document describing and reporting on the evidence collected. Data published at this stage of a research endeavor, thus represent a chance for immediate attribution. Most data repositories in Ecology and Evolution are primarily reviewed or checked for technical correctness and thus ensure that sharing is a viable opportunity for communicating findings in this form and recognizing participants in this process. Collectively, these latter three benefits highlight a moment in the scientific process to address more expeditious sharing and new ways to think about data publication as an instrument to shift the culture of science.

## 3 IMPLICATIONS

Now, could someone else find your data and use it? Sure. However, they are unlikely to write the same paper(s), one may or may not write about the work. Furthermore, reuse is preferable and a positive outcome. There are many solutions in place both at journals and within our scientific culture to protect and attenuate the risks and potential costs of sharing (Whitlock et al., 2016). Balance and fairness including collaboration with those that collect key components of data in addition to reasonable embargo periods still enable open science. The nuances of Ecology and Evolution and perhaps many disciplines that use a wide palette of experimental and synthesis tools likely limit the capacity for direct experimental replication from a dataset without additional details of the methods (Alston & Rick, 2021). Nonetheless, others can and will find a new use for the data. So, there is not zero risk to us of being scooped, but one does risk a lost chance for attribution and provenance for ideas (and people) embodied in the collection of those data. A critical implication is that we are often obligated by funders to publish data, and as described above, lags in peer review can be a real challenge and publication may not always be synchronous with the grant reporting cycle. Writing metadata does consume time that is unavailable for other activities such as writing the paper, but we are likely to commit the time at some point to satisfy requirements of agencies. Returns are also potentially higher in net efficiency and in recognition and reuse that benefit the researcher and team in having more to report. The main implication is that publishing sooner likely simplifies and makes your scientific life easier. It can support more efficient, rapid, and open work. So, if can consider it for some of your data some of the time, make a change and accelerate the research, recognition, and reuse process. A little bit more speed and openness will ensure that the early bird gets the return.

## CONFLICT OF INTEREST

There are no conflicts of interest.

## AUTHOR CONTRIBUTION

**Christopher J. Lortie:** Conceptualization (lead); Funding acquisition (lead); Methodology (lead).

### OPEN RESEARCH BADGES

This article has been awarded Open Materials, Open Data Badges. All materials and data are publicly accessible via the Open Science Framework at Data at figshare: https://doi.org/10.6084/m9.figshare.14773506.v1; CODE at Zenodo: https://doi.org/10.5281/zenodo.4959185, https://zenodo.org/record/4959185#.YMjrbC2cZTY

## Data Availability

Dataset: https://doi.org/10.6084/m9.figshare.14773506.v1 (Lortie, 2021)
